# Partially oxidized polyvinyl alcohol conduitfor peripheral nerve regeneration

**DOI:** 10.1038/s41598-017-19058-3

**Published:** 2018-01-12

**Authors:** Elena Stocco, Silvia Barbon, Lucia Lora, Francesca Grandi, Leonardo Sartore, Cesare Tiengo, Lucia Petrelli, Daniele Dalzoppo, Pier Paolo Parnigotto, Veronica Macchi, Raffaele De Caro, Andrea Porzionato, Claudio Grandi

**Affiliations:** 1 Section of Human Anatomy, Department of Neurosciences, University of Padua, Via Gabelli 65, 35121 Padua, Italy; 2 Department of Pharmaceutical and Pharmacological Sciences, University of Padua, Via Marzolo 5, 35131 Padua, Italy; 3 Department of Women’s and Children’s Health, Pediatric Surgery, University of Padua, Via Giustiniani 3, 35121 Padua, Italy; 4 Department of Plastic and Reconstructive Surgery, Ospedale Sant’Anna, Via Napoleona 60, 22100 Como, Italy; 5 Clinic of Plastic Surgery, Department of Neurosciences, University of Padua, Via Giustiniani 2, 35128 Padua, Italy; 6 Foundation for Biology and Regenerative Medicine, Tissue Engineering and Signaling (TES) ONLUS, Via De Sanctis 10, Caselle di Selvazzano Dentro, 35030 Padua, Italy

## Abstract

Surgical reconstruction of peripheral nerves injuries with wide substance-loss is still a challenge. Many studies focused on the development of artificial nerve conduits made of synthetic or biological materials but the ideal device has not yet been identified. Here, we manufactured a conduit for peripheral nerve regeneration using a novel biodegradable hydrogel we patented that is oxidized polyvinyl alcohol (OxPVA). Thus, its characteristics were compared with neat polyvinyl alcohol (PVA) and silk-fibroin (SF) conduits, through *in vitro* and *in vivo* analysis. Unlike SF, OxPVA and neat PVA scaffolds did not support SH-SY5Y adhesion and proliferation *in vitro*. After implantation in rat model of sciatic nerve transection, the three conduits sustained the regeneration of the injured nerve filling a gap of 5 mm in 12 weeks. Implanted animals showed a good gait recovery. Morphometric data related to the central portion of the explanted conduit interestingly highlighted a significantly better outcome for OxPVA scaffolds compared to PVA conduits in terms of axon density, also with respect to the autograft group. This study suggests the potential of our novel biomaterial for the development of conduits for clinical use in case of peripheral nerve lesions with substance loss.

## Introduction

Peripheral nerve injury is a common clinical problem significantly affecting the patients’ quality of life^1^. In case of severe transections, bridging of the gap between the proximal and distal nerve stumps is required^2,3^ and autologous nerve grafts using sensory nerves (i.e. the sural nerve or antebrachial cutaneous nerve) are the current criterion standard^4^. Nevertheless, donor-site morbidities (i.e. scarring and neuroma formation, permanent loss of function), size mismatch between the donor nerve and the injured one, poor functional recovery rates and increase in surgery and anesthesia time, have prompted the interest towards the identification of surgical alternatives to this technique^5–7^. Currently, the primary option is the use of hollow nerve guide conduits that will create an adequate microenvironment for nutritional support, axon regeneration and acting as a barrier against the surrounding tissue infiltration^8,9^. Autologous non-nerve tissues (i.e. bone, artery, vein, or muscle) have been widely used for this purpose; however, they are not devoid of disadvantages, therefore surgeons and researchers turned their attention towards different grafts/conduits made of biological or artificial polymers^10^. Natural biopolymers (i.e. chitosan, collagen, gelatin, hyaluronic acid, and silk fibroin) show high biocompatibility, biodegradability and interesting bioactive properties; nevertheless, they have some limitations like batch-to-batch variations and high costs due to the need of extensive purification and characterization^11^. Over time, the material choice for nerve conduits shifted towards biocompatible synthetic polymers^12^. Non-biodegradable polymers, such as methacrylate-based hydrogels, polyols (polyvinyl alcohol - PVA), polystyrene, silicone, and poly(tetrafluoroethylene), as well as biodegradable polyesters, such as poly(lactic acid) (PLA), poly(glycolic acid) (PGA), poly(lactic acid-co-glycolic acid) (PLGA), poly(ε-caprolactone) (PCL), polyurethanes, tri-methylene carbonate-co-ε-caprolactone, poly(D,L-lactide-co-ε-caprolactone) (PLCL) were used as nerve conduit materials^13–17^. To date, among these, the United States Food and Drug Administration (FDA) approved for clinical use nerve conduits made of PGA (NeuroTube, degradation time (*dt*): 3 mo), type I collagen (Neuragen, *dt*: 3–4 yrs; NeuroFlex, *dt*: 4–8 mo; NeuroMatrix, *dt:*4–8 mo; NeuraWrap, *dt*: 36–48 mo; NeuroMend, *dt:* 4–8 mo); PLCL (Neurolac, *dt*: 16 mo); PVA (SaluTunnel, *dt*: Non-biodegradable); processed human nerve allograft (Avance); extracellular matrix derived from porcine small intestine submucosa (AxoGuard, *dt*: No data)^18^.

In the light of this, the purpose of the present study was to ascertain, through *in vitro* and *in vivo* studies, the effectiveness of a nerve conduit made of 1% Oxidized PVA (OxPVA), a novel resorbable biomaterial recently developed by our research group^19^. Thus, its performances were compared with that of neat PVA and Silk-Fibroin nerve guides. As mentioned above, PVA conduits are the only non-degradable synthetic nerve guides approved by the FDA; to our knowledge, however no information about the repair efficacy of PVA conduits has been reported in literature^20,21^. Concerning Silk-Fibroin, both *in vitro* and *in vivo* studies have demonstrated its good potential in the reconstruction of peripheral nerves; nevertheless, neither the FDA nor any other administration has approved any silk conduit in clinical practice^18,22^.

## Results

### Fabrication and morphological characterization of scaffolds

**Figure 1:**
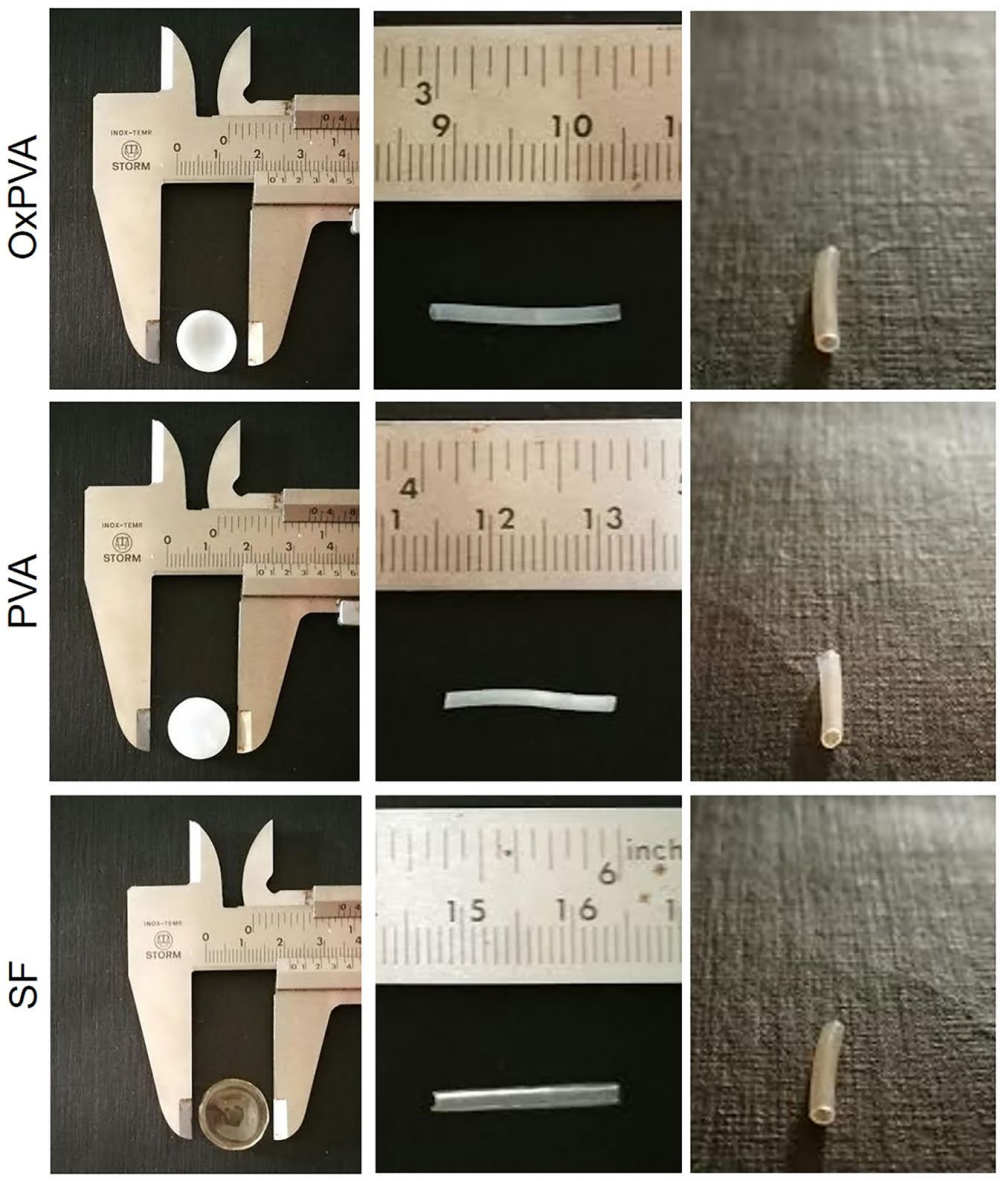
Gross appearance of scaffolds. Gross appearance of disk-shaped and tubular scaffolds made of OxPVA, PVA and SF.

After the manufacture of the scaffolds, a morphological characterization was first performed. In Fig. 1 the gross appearances of both disk-shaped and tubular scaffolds can be appreciated. Considering PVA-based supports, they were dimmed when in the shape of disks; that was dependent on the volume of polymer poured into the mold. Conversely, SF disk-shaped scaffolds were completely transparent. Tubular scaffolds were all highly transparent and the lumen was clearly recognizable in all samples.

### *In vitro* cell growth on scaffolds

**Figure 2:**
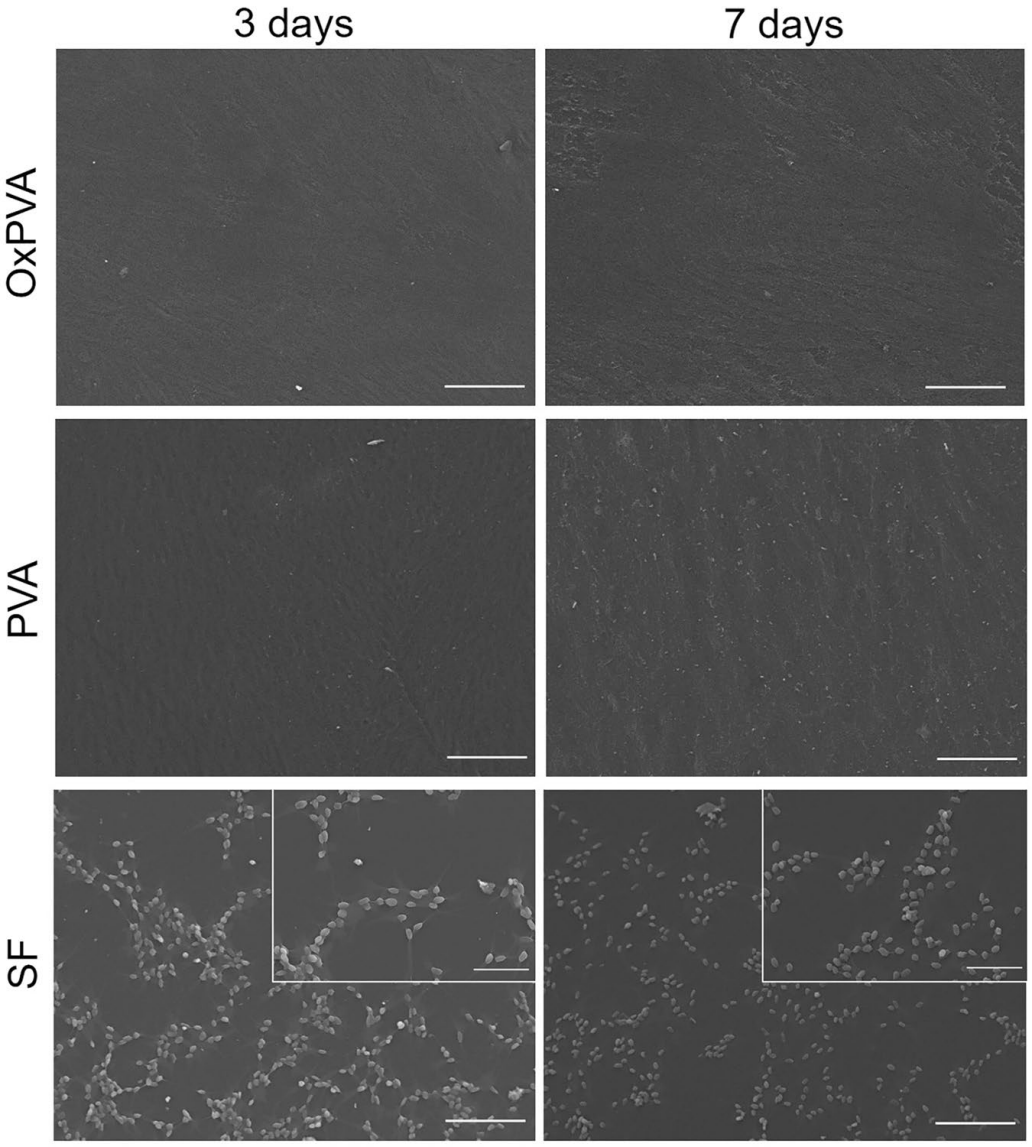
Assessment of cell adhesion and proliferation on scaffolds. SEM micrographs of SH-SY5Y cells after 3 and 7 days from seeding on scaffolds (scale bars = 100 µm; scale bars in upper right inserts = 50 µm).

The biologic activity of the biomaterials was preliminarily evaluated *in vitro* using a human-derived neuroblastoma cell line (SH-SY5Y). According to Scanning Electron Microscope (SEM) micrographs (Fig. 2), both PVA-based scaffolds did not promote cell adhesion and proliferation as no cells were detectable on the supports at the two end-points considered. Conversely, SF scaffolds favorably sustained SH-SY5Y growth and cells proliferation. Even though SH-SY5Y did not form a monolayer, they appeared randomly distributed on SF scaffolds with a typical spindle-elongated morphology. The 3-(4,5-dimethylthiazol-2-yl)-2,5-dimethyltetrazolium bromide (MTT) assay confirmed SEM analysis results; PVA-based scaffolds did not sustain cell growth in comparison with SF supports (p < 0.01) which also showed a progressive increase of cell number from day 3 to day 7 (p < 0.01).

### Surgical procedure: implantation and removal

**Figure 3:**
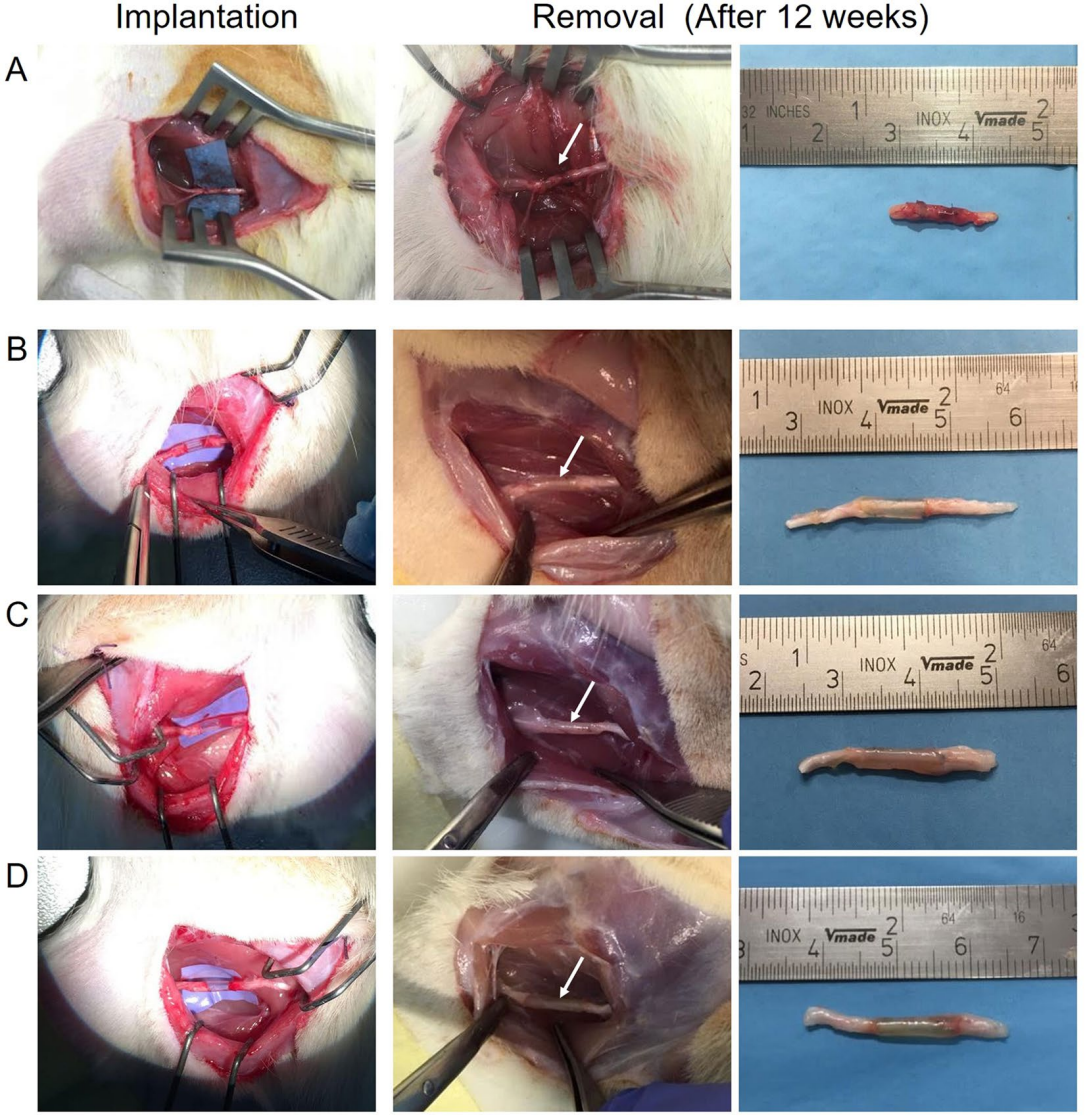
Intraoperative photographs of reversed-autograft and nerve conduits implantation and removal. Implantation and removal of reversed-autograft (**A**); OxPVA (**B**); PVA (**C**) and SF (**D**) nerve conduits in a rat sciatic nerve transection model. Twelve weeks after surgery, the prostheses were preliminarily compared for their gross appearance at the implant site (white arrow) prior to explant.

At the time of implantation, PVA-based nerve conduits showed certain flexibility in comparison with SF scaffolds. Nevertheless, all of them were easy-suturing, demonstrating an adequate tear-resistance feature. The transparent appearance of the guides allowed to verify that the gap left between the stumps was appropriate (Fig. 3). The incisions showed no signs of infection and healed adequately approximately one week after surgery. All animals involved in the experimental study tolerated the anesthesia/surgery and survived until the end of the observation period.

Three weeks after surgery, animals, including that of the reversed-autograft group, showed the tendency to develop ulcers on the left plantar; lesions were treated with the systemic administration of antibiotics/analgesics and healed in few days. At 12 weeks post-implantation, the animals were euthanized. During dissection, all nerve conduits were still clearly recognizable; they were encapsulated in an extremely thin fibrous tissue, highlighting that the host tissues tolerated the implanted conduits without eliciting foreign body reactions. Moreover, visual inspection after surgery showed that all nerve conduits preserved their round shape as well as a uniform diameter along the entire length of the implant. As regards the animals implanted with the reversed-autograft, the segments were recognizable.

At the time of explantation, all tubes appeared well integrated into the host tissue and no dislocation was observed; furthermore, no inflammatory reaction occurred demonstrating that implants were biocompatible with the peripheral nerve tissues. Even neuroma formation was not observed at the proximal or distal coaptation site in any of the rats. Considering the reversed-autograft group, no complications were observed as guaranteed by the autologous nature of the implanted segment.

Examination by surgical microscopy of explanted tubes, confirmed neo-tissue formation in all experimental groups. The high transparency of conduits, allowed to directly see the presence of a regenerated tissue throughout the lumen of the implants appearing as a white tubular substance passing along the conduit.

### Assessment of axonal regeneration

**Figure 4:**
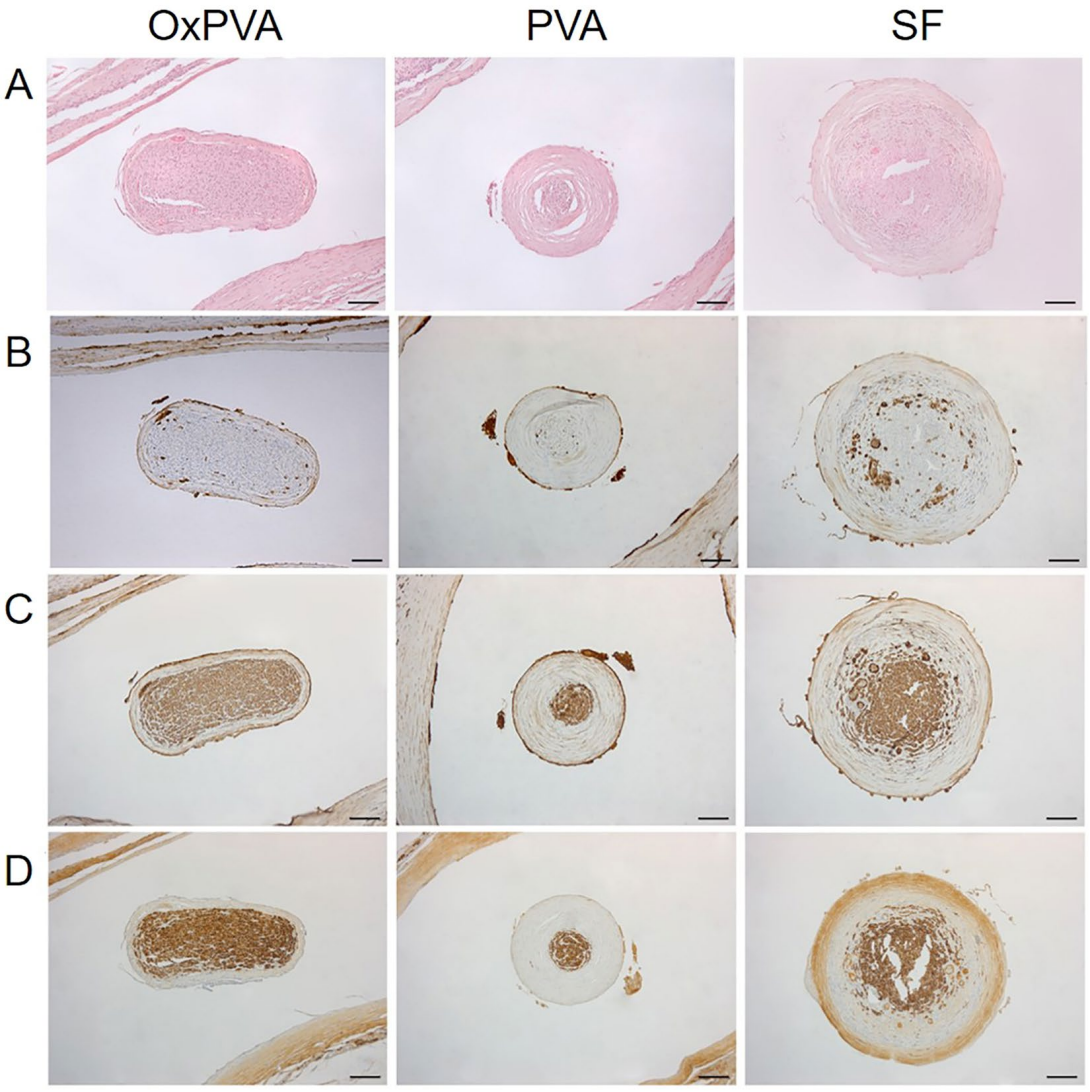
Histological and immunohistochemical analysis. Characterization of the central portion of explanted OxPVA, PVA and SF grafts by HE staining (**A**) and immunohistochemical anti-CD3 (**B**), anti-β-tubulin (**C**) and anti-S100 (**D**) reactions (scale bar = 100 µm).

Axonal regeneration through the nerve conduits was compared with that assured by the implant of an autologous segment of nerve (reversed-autograft – control group; see Supplementary Fig. S1). Hematoxylin and eosin (HE) staining of the central portion of each explanted tube showed that the regenerated nerve successfully grew through the gap connecting proximal and distal nerve stumps (Fig. 4A). In all groups, the presence of an external fibrous layer which envelopes the biomaterial was clearly identifiable. Regenerated nerve was clearly recognizable inside the nerve guides. Moreover, OxPVA nerve conduits promoted the formation of a regenerated nerve characterized by a thinner and more compact perineurium than PVA and SF guides. Anyhow, the neo-formed tissue of each section appeared in the middle dense and well organized, comparable to that of the reversed-autograft group (see Supplementary Fig. S1).

HE staining did not show severe inflammatory infiltrates in any sample. Anti-CD3 immunohistochemistry demonstrated that severe inflammatory reactions were not present in all the samples apart from a very slight infiltration of the connective tissue surrounding the implanted material (Fig. 4B). These data further demonstrated that the host tissue tolerated the implanted conduits. As regards the reversed-autograft group, CD3 positive cells were not detected, due to the autologous origin of the graft (See Supplementary Fig. S1).

Axonal regeneration into the constructs was assessed by β-tubulin (Fig. 4C) and S100 (Fig. 4D) staining which highlighted that the three nerve-conduits permitted the reconnection of proximal and distal stumps. Moreover, the reversed-autograft group showed a strong positivity for both β -tubulin and S100 (See Supplementary Fig. S1).

**Figure 5:**
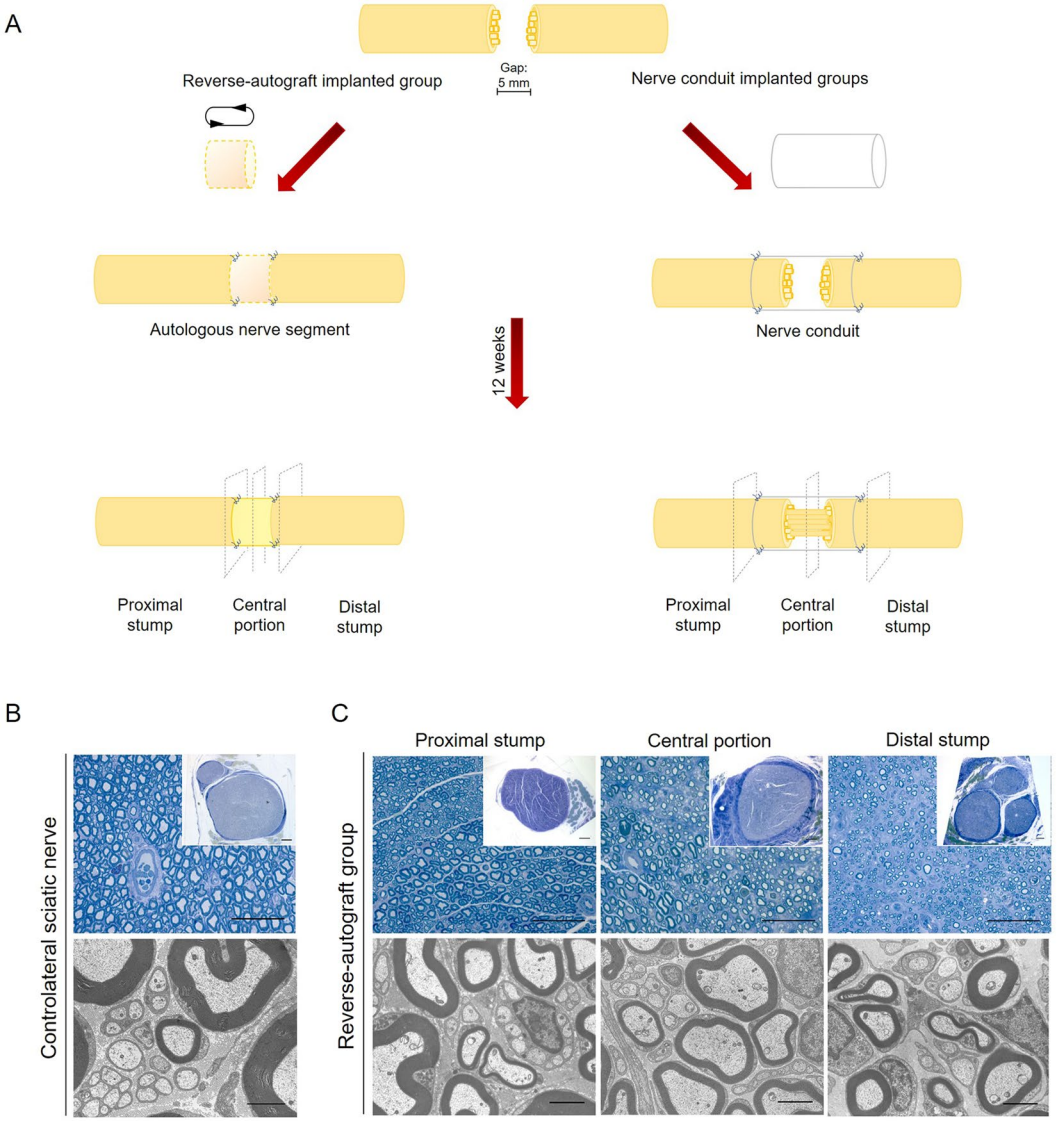
Experimental design and representative evaluation by Toluidine Blue and TEM of the contralateral and reversed-autograft at 12 weeks from surgery. (**A**) Schematic illustration of the experimental design, consisting in entubulating the proximal and distal stump of the transected sciatic nerve (gap = 5 mm) by a reversed-autograft or a conduit which has to guide the regeneration of nerve fibers. At 12 weeks from surgery, samples were excised and divided into 3 sections (proximal stump, central portion and distal stump) according to transverse planes each of which was analyzed. (**B**) Representative Toluidine Blue staining image (scale bar = 50 µm; scale bar in upper right insert: 100 µm) and TEM micrograph (scale bar = 2 µm) of the controlateral sciatic nerve of a Sprague-Dawley rat. (**C**) Toluidine Blue staining (scale bar = 50 µm; scale bar in upper right insert: 100 µm) and TEM micrographs (scale bar = 2 µm) of the proximal, central and distal section of a reversed-autograft at 12 weeks from surgery.

The explanted OxPVA, PVA and SF nerve conduits were investigated according to the experimental design reported in Fig. 5A. In Fig. 5B,C is showed the appearance of both the contralateral sciatic nerve and the reversed-autograft segment at 12 weeks from surgery of Sprague-Dawley rats. The samples were stained with Toluidine Blue and observed at Transmission Electron Microscope (TEM). In both, the typical peripheral nerve morphology was clearly recognizable. Externally there was the perineurium, the connective-tissue sheath that surrounds nerve fibers. Hence, homogeneously distributed myelinated and unmyelinated axons were identifiable in the middle.

**Figure 6:**
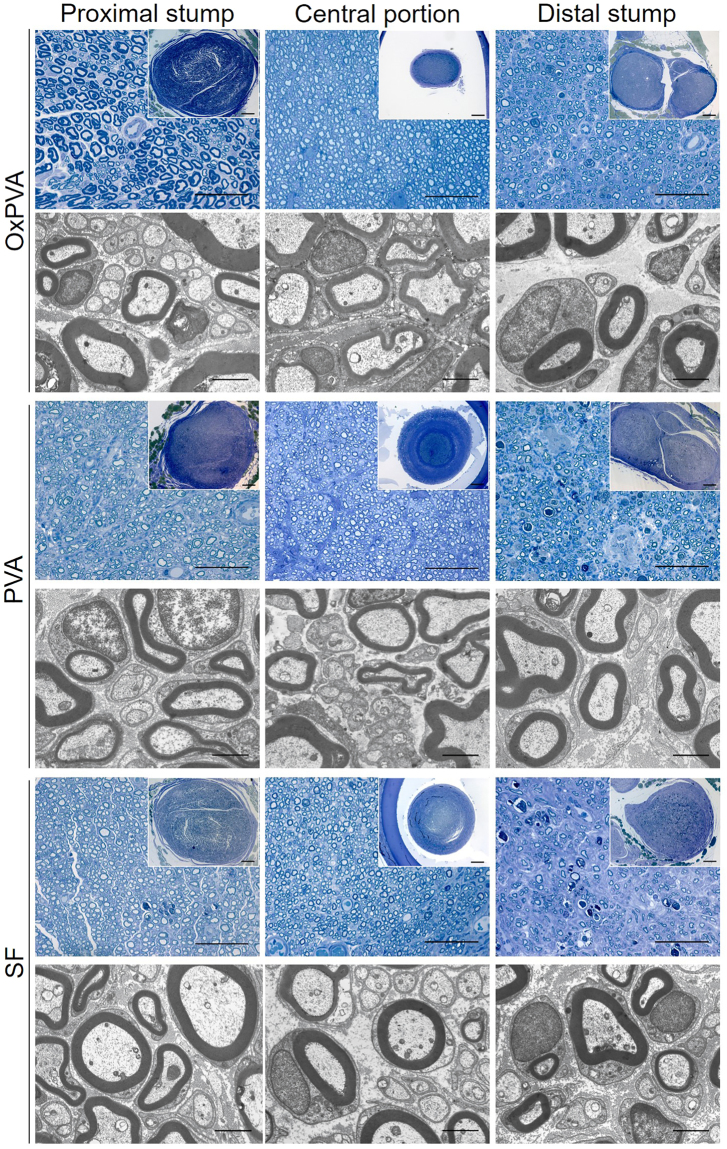
Evaluation of Sprague-Dawley sciatic nerve characteristics at 12-weeks after surgery. Cross-sections of the proximal, central and distal portion of rat sciatic nerve transection models implanted with OxPVA, PVA and SF conduits. Samples were evaluated with Toluidine Blue staining (scale bar = 50 µm; scale bar in upper right insert = 100 µm) and TEM (scale bar = 2 µm).

Considering the central portion, all nerve conduits could support nerve regeneration, as newly-formed axons (myelinated and unmyelinated) were identifiable in all sections. Nevertheless, some differences were found between the groups. OxPVA explants showed a regenerated nerve characterized by a thinner perineurium than PVA and SF implanted animals (Fig. 6). In particular, SF scaffolds developed the formation of a thick and fibrotic perineurium. Moreover, OxPVA and PVA showed a regenerated nerve characterized by compartmented myelinated and unmyelinated nerve fibers as demonstrated by the presence of septa. Conversely, septa were not visible in the central portion of SF nerve conduits which, on the contrary, showed the presence of a fibrotic matrix surrounding axons. In parallel, the tissue-quality of the proximal and distal stumps was also considered. The proximal stump of OxPVA samples appeared different from the others as myelinated axons were larger and more similar to that of the controlateral sciatic nerve. Many small, newly-regenerated axons were also identifiable. Degenerated axons were recognizable in the proximal and distal stump of SF implanted nerves, as well as in the distal stump of PVA-implanted samples. In particular, TEM images showed the presence of many new-formed myelinated and unmyelinated nerve fibers in all samples, as well as Schwann cells surrounding myelinated axons. Compared with the contralateral sciatic nerve (Fig. 5B), the newly regenerated myelinated axons had a thinner myelin sheath; nevertheless, the morphology of new axons was normal and adequate; the appearance of the neo-regenerated tissue was more similar to that of the reversed-control group (Fig. 5C).

**Figure 7:**
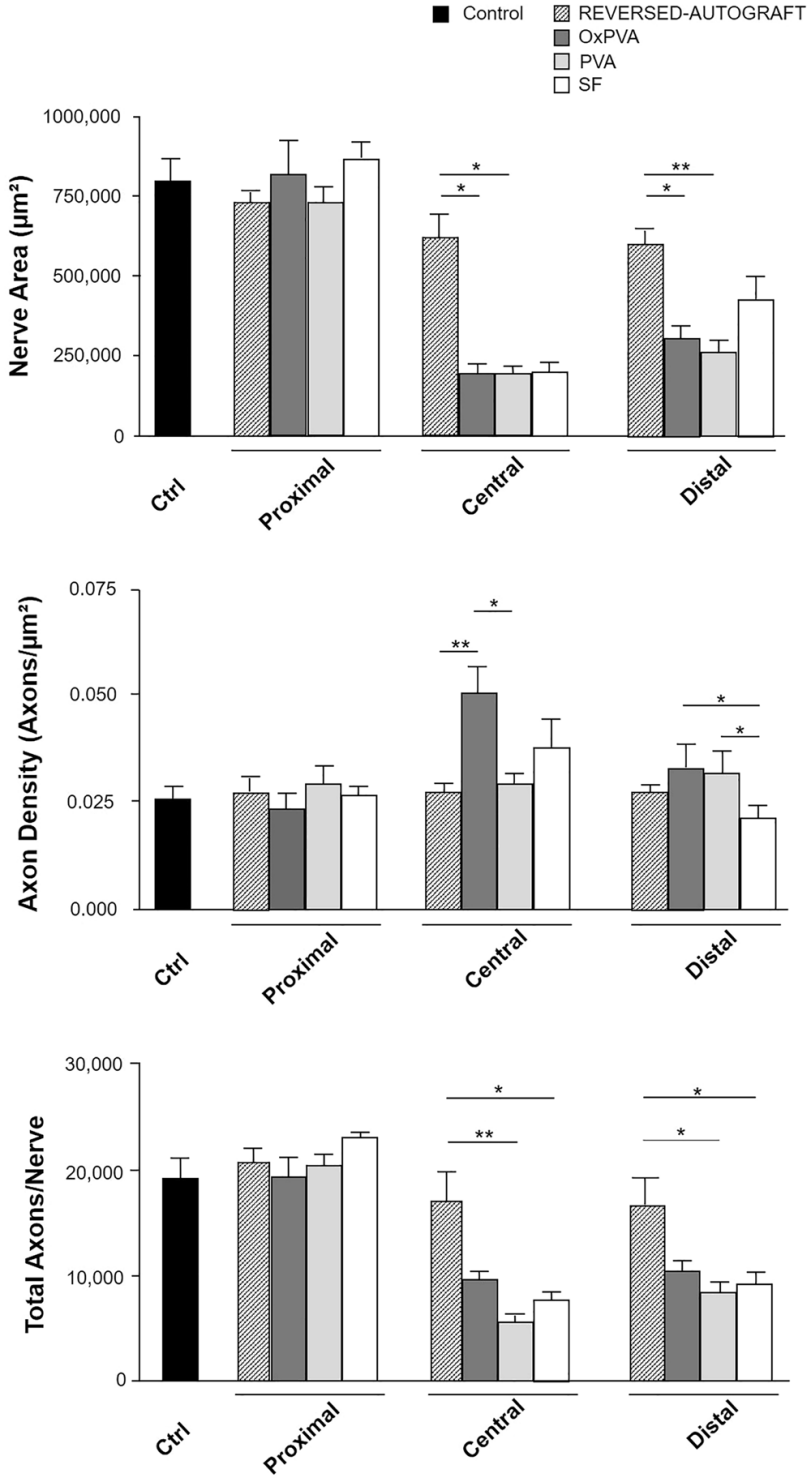
Morphometric assessment of regenerated sciatic nerves at 12-weeks after surgery. Histograms show mean nerve area (µm^2^), axon density (axons/µm^2^) and total axons/nerve of the proximal, central and distal portions of reversed-autograft, OxPVA, PVA and SF explanted samples versus native sciatic nerve. Statistical analyses were performed by the Kruskal-Wallis test and Dunn’s multiple comparison test. Results are expressed as mean values ± SD (^*^p < 0.05; ^**^p < 0.01).

Morphometric data of the regenerated sciatic nerves were represented in the histograms; data were expressed as mean value ± standard deviation (SD) (Fig. 7).

Comparing the nerve areas, significant differences were found at the central and distal level. In particular, as regards the central portion, reversed-autograft mean area (617,848 ± 72,207 µm²) was significantly higher than the mean areas of OxPVA (195,248 ± 29,061 µm², p < 0.05) and PVA (191,485 ± 28,154 µm², p < 0.05), respectively. PVA, OxPVA and SF groups showed a mean nerve area value which corresponded to the 31.6%, 31% and 32.3% respectively, of the reversed-autograft group one.

Also at the distal level, reversed-autograft showed a significantly higher mean area (595,848 ± 43,765 µm²) with respect to OxPVA (307,627 ± 42,292 µm², p < 0.05) and PVA (263,131 ± 38,817 µm², p < 0.01); otherwise, no difference was highlighted with respect to the SF group (429,029 ± 76415 µm²).

OxPVA showed a higher Axon Density (0.0496 ± 0.007 axons/µm²) than PVA (0.0282 ± 0.004 axons/µm², p < 0.05) and reversed-autograft (0,0274 ± 0.004 axons/µm², p < 0.01) in the central portion of the samples. In the distal stump, both OxPVA (0.0332 ± 0.005 Axons/µm², p < 0.05) and PVA (0.0318 ± 0.005 Axons/µm², p < 0.05) showed significantly higher Axon Density than SF (0.0208 ± 0.003 Axons/µm²). There was no significant difference between conduits and reversed-autograft (0.0278 ± 0.004 Axons/µm²).

In the central portion, reversed-autograft showed higher Total Axons/Nerve (16,863 ± 2,628) than PVA (5,455 ± 1,375 p < 0.01) and SF (7,603 ± 1,571, p < 0.05); no significant difference was present between reversed-autograft and OxPVA (9,579 ± 1,184). Also in the distal section differences were detected between the reversed-autograft (16,545 ± 2,519) and the SF (9,081 ± 2,878, p < 0.05) as well as the PVA (8,391 ± 2,039 p < 0.05); in this segment, the reversed-autograft assured better results in term of total axons/nerve in comparison with PVA and SF; however, significant differences were not highlighted with respect to the OxPVA group (10,277 ± 2,391).

### Functional assessment of nerve regeneration

**Figure 8:**
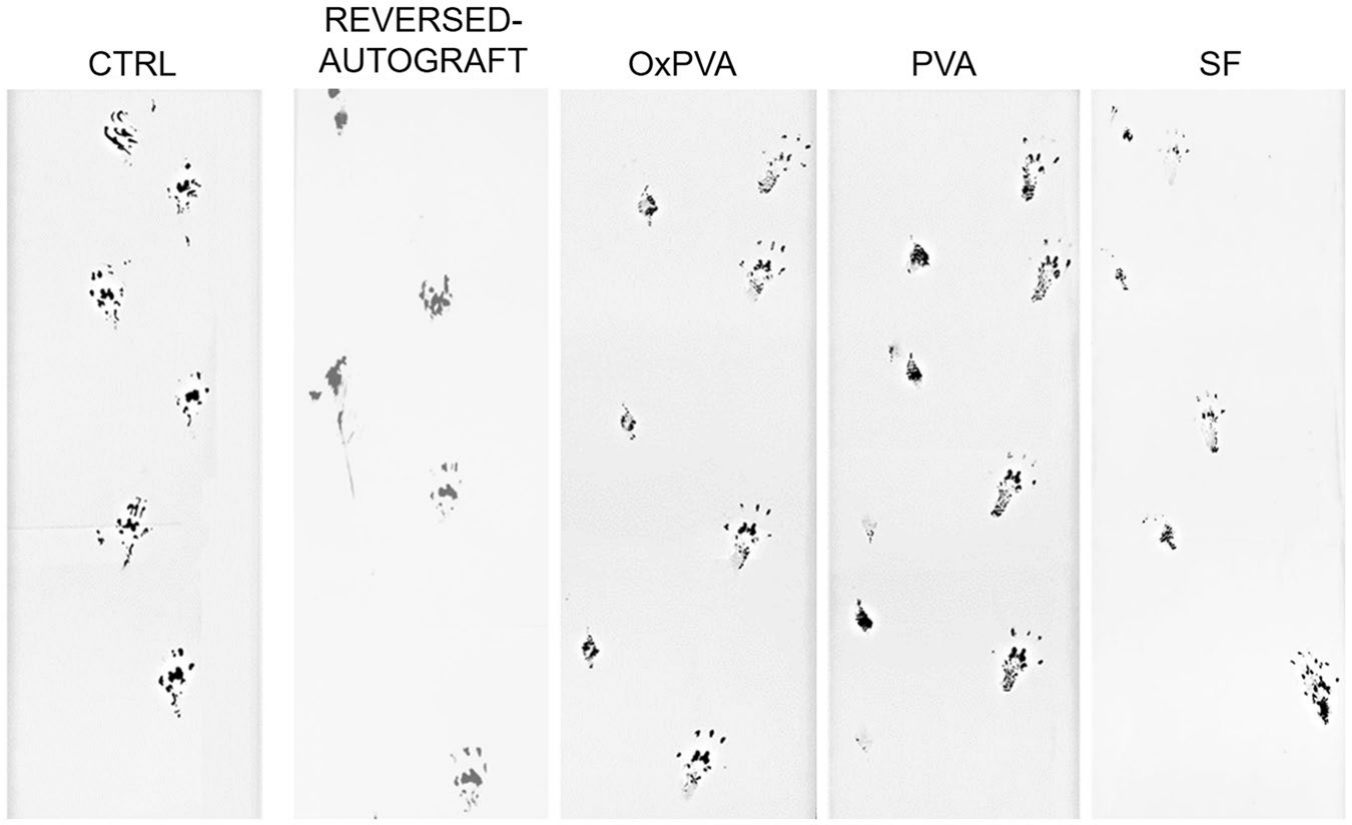
Footprints analysis. Representative footprints analysis performed before (ctrl) and 12 weeks after left sciatic nerve transection and implantation of a reversed-autograft, OxPVA, PVA and SF nerve conduits.

Prior to euthanizing the animals, the functional recovery was assessed by the gait analysis (See Supplementary video S2). All animals supported their body weight on the hind leg; however, as the implanted animals showed a tendency for auto-mutilation of operated limb digitis, the analysis resulted more complex. As a consequence, it was possible to evaluate only the qualitative appearance of trails in comparison with non-operated Sprague-Dawley rats and the reversed-autograft group; the contralateral footprint was considered as internal control. At 12 weeks from surgery, the animals showed a gait recovery; however, the records showed a worse function of paw in PVA group than the others as the footprints appeared closer and limping (Fig. 8).

## Discussion

Autografts are the gold standard for failed primary nerve repairs but the resulting donor-site morbidity represents a significant issue. Hence, along with decellularized allografts, nerve conduits are a novel and interesting solution^23^. As full regeneration after nerve injury remains one of the principal goals of regenerative biology, many biopolymers and their composites/derivatives have been used to develop nerve guides^24^; however, each has its own limitations^25^. In the present study, a novel synthetic and biodegradable biomaterial was exploited to obtain a nerve conduit (i.e. PVA with an oxidation degree of 1%). In particular its specific properties were compared with those of the non-biodegradable PVA counterpart and the natural biopolymer SF.

The assessment of the interplay between cells and the supports represents a key step to evaluate *in vitro* the potential for neural regeneration of biomaterials^26^, offering information regarding cytotoxicity, genotoxicity, cell proliferation and differentiation^27^. Our analyses led to confirm that, unlike SF, PVA-based supports were not capable of sustaining SH-SY5Y cells proliferation. Anyhow, in our experimental conditions, the lack of cell adhesion was not due to any cytotoxic properties of the material (data also confirmed by the *in vivo* study). In fact, PVA hydrogels have been proposed for a number of biomedical and pharmaceutical applications (i.e. heart valves, corneal implant, cartilage substitute and arterial phantoms) and among its advantages the non-toxicity stands out. Cell adherence on PVA hydrogels is inhibited by its highly hydrophilic nature^28^, as previously demonstrated also for the OxPVA^19,29^.

Even though PVAs showed lower adhesion properties to SH-SY5Y cells, *in vivo* experimental approaches demonstrated good nerve regeneration with all the types of nerve guides. It is possible that the low adhesion properties of PVAs also with respect to other cell types, such as fibroblasts^29^, may have favored axon growth, preventing the formation of fibrous tissue.

According to the literature, the beneficial characteristic of synthetic materials compared to natural ones is that the degradation rate and mechanical properties can be controlled, creating an opportunity to individualize the patient’s treatment^5^. As such, OxPVA conduits potential resides in the versatility of the chemical reaction required to prepare the polymeric solution. Oxidation provides for the introduction of carbonyls in the PVA backbone which decreases the number of crosslinkable groups/chain; hence, the resulting crosslinked hydrogel will show reduced mechanical properties (stress at break/strain at break) which may be counterbalanced by increasing the number of freezing-thawing (FT) cycles. By modulating the content of carbonyls and the amount of crosslinking cycles it is possible to improve or reduce the tear resistance of the polymer, customizing the final product according to the needs of the Manufacturer^19,30^. Here, OxPVA nerve conduits were prepared using a polyvinyl alcohol solution with an oxidation degree of 1%. Greater oxidation rates (i.e. 2%) would not have allowed to obtain a manipulable and suturable nerve guide. Moreover, to ensure a prolonged stay of the nerve guide in the surgical site, the number of crosslinking FT cycles was preliminarily calibrated considering that the minimum degradation time for commercially available nerve conduits is 3 months^18^.

Animal models are essential for evaluating biocompatibility, tissue response and mechanical function of any nerve conduit^27^. Because of its size, the rat sciatic nerve has been the most commonly used experimental model in studies which consider peripheral nerves regeneration and the development of new nerve guides^24^. The characteristics that an optimal conduit for nerve regeneration has to satisfy are adequate elasticity and resistance to suture. All the investigated nerve guides responded to these requirements, even though Ox PVA nerve guides gave better results. Moreover, a particularly good surgical handling was guaranteed by OxPVA tubes followed by PVA and SF ones; these last guides showed a major stiffness ascribable to robust mechanical properties of silk^31^. Considering the surgical site, it is preferable to have tubes with a certain elasticity and capable of supporting the movement without collapsing; these requirements were more satisfied by PVA-based conduits than SF ones.

The hollow tubes remained in the site of injury for 12 weeks during which the behavior of animals was carefully monitored. A variety of approaches has been used in literature to access animal functional recovery and neural tissue repair after sciatic nerve injuries; among these, footprint analysis is a widely used non-invasive method to gain data about the sciatic functional index (SFI)^32^. In accordance with other Authors, a certain inclination to digitis auto-mutilation was observed^33,34^, partially affecting the evaluation of functional recovery of the injured sciatic nerve. This feature was noticed in the reversed-autograft group as well, suggesting the absence of a specific correlation with the implant of a hollow tube. However, considering the operated limb, the distances between fingerprints were wider in OxPVA, SF and in the reversed-autograft implanted animals than in PVA, suggesting the absence of painful symptoms.

At 12 weeks from surgery, nerve guides were all clearly identifiable in the implant site confirming that seven FT cycles allowed to get a guide with biodegradation characteristics in line with those of commercial products; moreover, no dislocation occurred. Also, the autologous segment was still recognizable. This data confirms the capability of the three types of conduit to persist for a sufficient period to allow the formation of a fibrin matrix to connect the proximal and the distal nerve stumps as well as an adequate mechanical strength to resist *in vivo* physiological loads like muscular contraction. In parallel, the absence of inflammatory exudates, abscesses and neuromas pointed out the biocompatibility of the biomaterials as well as their adequate interaction with the nerve stumps, excluding the occurrence of issues related to swelling that can occlude the inner lumen, inappropriate degradation rates, and cytotoxic degradation products which are believed to be associated with inhibiting regeneration^35^.

In most of the literature, the regeneration of nerve fibers could not be directly appreciated because the conduits are not transparent. In those cases, the guides must be cut open for the observation of the regenerated nerve^36^. Conversely, the investigated tubular scaffolds are transparent so that they can permit to confirm the success of nerve regeneration *in situ*. The immediate verification of the presence of a regenerated tissue inside the tubes suggested that they provided correct guidance for tissue growth. However, its nature was confirmed with certainty by further histological and immunohistochemical analysis which, in accordance with the results obtained by the reversed-autograft group, highlighted the positivity for the specific markers for axons and Schwann cells (i.e. β-tubulin and S100, respectively)^37^.

Moreover, histologic and TEM analysis related to the central portion of explants of each experimental group highlighted the presence of numerous small neo-regenerated nerve fibers (myelinated and unmyelinated axons); while, considering proximal and distal sections, an amorphous fibrotic matrix was clearly identifiable among the axons; similar features were observed also in the reversed-autograft group. As concerns the proximal stump, fibrosis may be ascribed to Wallerian degeneration process. In 12-weeks the re-connection between the two stumps occurred in all three cases suggesting the efficacy of the implanted nerve conduits. An effective reconjunction was also assured by the autologous segment. Considering distal stumps, a reduction in size in comparison with the proximal ones was present in all the groups. The lack of direct contact with proximal neurons causes a reduced expression of neurotrophic growth factors, changes in the extracellular matrix and loss of Schwann cell basal lamina. All these events hamper axonal extension^38^. The mean area of the central portion of OxPVA, PVA and SF samples was comparable, except for the reversed-autograft group showing a significantly higher mean area with respect to the OxPVA and PVA. Interestingly, the tree hollow conduits assured the regeneration of a similar size tissue area corresponding to the 31.6% (OxPVA), 31% (PVA) and 32.3% (SF) of the reversed-autograft one. Significant differences were also found between reversed-autograft and PVAs in the distal segment. Analyzing data on axon density, there were significant differences in both the central and distal sections. In the central portion, OxPVA nerve conduits guaranteed for a significantly higher density of axons than the reversed-autograft and the PVA, respectively. In the distal segments, both PVAs assured a better outcome in comparison with SF; there was no significant difference between conduits and reversed-autograft.

The total number of counted axons/nerve in the central portion was significantly higher in reversed-autograft than PVA and SF; interestingly, no significant difference was detected between reversed-autograft and OxPVA as well as comparing the OxPVA, PVA and SF conduits. Considering the distal segment, the reversed-autograft group assured better results in term of total axons/nerve with significant differences in comparison with PVA and SF (p < 0.01) but not with OxPVA. Therefore, based on the morphometric analysis, two considerations can be made regarding the performance of the OxPVA conduits at the central and distal level. Although in the central portion the nerve area of OxPVA was significantly inferior to that of the reversed-autograft, axon density resulted significantly higher; moreover, even if the total number of axons of OxPVA was lower than that of the reversed-autograft, the value was not statistically significant. In the second instance, albeit in the distal segment the area of OxPVA was significantly lower to that of the reversed-autograft, the axon density and the total axons/nerve count were not significantly inferior with respect to the values assured by the reversed-autograft.

It has been described that within the first hours following nerve transection and conduit implantation, wound fluid rich in nerve-supporting factors fills from both nerve stumps into the conduit inner lumen. During this process, the fibrin matrix accumulates naturally to form a complete bridge across the gap providing a substrate for Schwann cells migration^35^. Interestingly, these differences which suggest a better effectiveness of OxPVA then PVA and SF in aiding axonal growth within the tubular prosthesis may be ascribed to the chemical oxidation of the polymer. In fact, the introduction of carbonyls on the PVA backbone supports the protein-loading ability of the scaffold due to possible Schiff-base interaction between the amino-groups of neurotrophic substances released by stumps and carbonyls of oxidized PVA^19,39,40^.

## Conclusions

According to the results of this study, all nerve conduits considered (i.e. OxPVA, PVA, and SF conduits) promoted peripheral nerve regeneration in case of neurotmesis with loss of substance. OxPVA nerve conduits could represent the development of PVA ones; in addition to an excellent degree of manipulability during surgery, elasticity and tear-resistance features, they assured nerve regeneration characterized by a homogeneous distribution of myelinated and unmyelinated nerve fibers and an axon density in the middle of the conduit significantly higher to that of reversed-autograft. The interesting *in vivo* outcome could be attributed to the chemical structure of the polymer as it would promote the protein-loading due to possible Schiff-base interactions between amino-groups of neurotrophic substances released by stumps and carbonyls of oxidized PVA.

Further studies will consider the functionalization of OxPVA nerve conduits with neurotrophic factors.

## Methods

### Preparation of the polymer solutions

OxPVA (1% oxidized PVA) was developed by a method we patented based on a controlled chemical oxidative reaction^19,41^.

Regarding PVA solution, it was obtained by suspending in MilliQ water a certain quantity of PVA powder (Mw: 146,000–186,000 Da, 99+% hydrolyzed) and heating the suspension until complete polymer dissolution (48 h at 100 °C under stirring).

Lastly, SF solution was set up from *Bombyx mori* silk cocoons according to a two-step protocol described by Rockwood and Collegues^42^. Small pieces of silk cocoons were degummed by boiling in a solution of sodium carbonate ([Na_2_CO_3_] 0,02 M) for 30 min, rinsed in distilled water to remove the sericin and dried overnight. Thereafter, the degummed silk was dissolved in a 9.3 M LiBr solution at 60 °C for 4 h, dialyzed extensively against deionized water using a cellulose membrane dialysis cassette (molecular cut-off 12,400 Da) and then centrifuged twice at 5–10 °C and 9,000 rpm for 20 min.

### Manufacture of scaffolds

Disk-shaped scaffolds were prepared using a 24-well tissue culture plate as mold. A certain volume of PVAs solutions were respectively poured in each well of the plate (0.4 ml/well) and crosslinked by freezing (at −20 °C for 24 h)/thawing (at 2.5 °C for 24 h) (FT). After seven FT cycles, the resulting hydrogels were stored at −20 °C until use. As for SF disk-shaped scaffolds, 0.4 ml of SF solution were poured in each well of the plate and dried over-night; thereafter, the scaffolds were incubated in 70% Methanol-Water for 2 h to allow the conversion of fibroin into a beta-structure.

Regarding nerve conduits, these were manufactured according to the injection molding-technique (PVA-based nerve conduits) or the mandrel-coating technique (SF nerve conduits). OxPVA and PVA polymeric solutions were poured in a tubular mold (internal diameter: 2.1 mm) equipped with a central coaxial mandrel (diameter: 1.2 mm); thereafter, FT occurred as previously described. About SF nerve conduits, a glass mandrel (diameter: 1.6 mm) was dipped into the solution to form a thin polymer layer; after drying for 30 min at room temperature (RT), six coating cycles were performed. Hence, solvent evaporation occurred over-night allowing the formation of the SF scaffold. This step was repeated six times before incubation in 70% Methanol-Water for 2 h. Thus, the silk cylinder was carefully removed.

All products were disinfected with 70% alcohol and washed with phosphate buffered saline (PBS 0.1 M, pH 7.4) prior to use.

### *In vitro* biocompatibility

A stabilized human neuroblastoma cell line (SH-SY5Y) was used to test *in vitro* the biological behavior of scaffolds. Cells were purchased by ECACC (European Collection of Cell Cultures, Porton Down, United Kingdom) and cultured in 75 cm^2^-flasks at 37 °C in humidified atmosphere containing 5% CO_2_. They were grown in DMEM/F-12 (1:1) basal medium added in 15% FBS, 1% non-essential amino acids and 1% antibiotic solution (penicillin/streptomycin). At a confluence of 80%, SH-SY5Y were detached using 0.25% Trypsin-EDTA and reseeded for expansion. Cells were daily observed by optical microscope.

After expansion, SH-SY5Y cells were detached and counted by a TC20 Automated Cell Counter. Hence, they were seeded (20,000 cells/cm^2^) on disk-shaped supports; cell adhesion/growth on scaffolds were evaluated by SEM and MTT assay at 3 and 7 days after seeding. Briefly, at each end-point, samples were fixed for 24 h in 2.5% glutaraldehyde in 0.1 M cacodylate buffer (pH 7.2) and dehydrated with a graded ethanol series. Thereafter, critical-point drying and gold sputtering were performed and finally samples were observed by SEM. Images were taken by JSM-6490 SEM (Jeol USA, Peabody, MA, USA). As regards MTT assay, cell cultures were treated with a MTT solution (0.5 mg/ml) for 4 h. Formazan precipitates were solubilized in 2-propanol acid (0.04 M HCl in 2-propanol) and the optical density was measured at 570 nm using a Microplate auto-reader EL 13 (BIO-TEK Instruments, Winooski, Vt., USA). Results were expressed as the number of cells grown on the seeded surface.

### Animal model and *in vivo* study

All animal procedures were approved by the ethical committee of Padua University, in agreement with the Italian Department of Health guidelines.

Forty Sprague-Dawley rats, weighing approximately 200–250 g, were randomly divided into four experimental groups (n = 10/each): reversed-autograft, OxPVA, PVA, and SF group and anaesthetized using a binary gas mixture of isoflurane/oxygen. Hence, prior to perform a 2.5 cm incision on the left thigh, the area was shaved and disinfected. After separating the fascia and muscle groups by blunt dissection, the left sciatic nerve was exposed and transected creating a nerve gap of 5 mm between the proximal and distal stumps. As regards the reversed-autograft group, a segment of sciatic nerve (5 mm in length) was excised, inverted and reimplanted using Nylon 8–0 sutures. Concerning the conduits (10 mm in length), these were coaxially interposed between the stumps and then sutured to the epineurium using Nylon 8–0 sutures. The incision was closed in layers using 4–0 silk sutures. After surgery, the animals were allowed to recover in the cage and they were treated with anti-inflammatory (Rimadil, 5 mg/kg) and antibiotic (Bytril, 5 mg/kg) therapy for 5 days. In the following period, they were housed in a temperature-controlled facility and were given laboratory rodent diet and water *ad libitum*.

After 12 weeks, the rats were euthanized by carbon dioxide asphyxiation and the implants were excised and preliminarily analysed for their size/integrity. Thereafter, samples were properly fixed for histological/immunohistochemical (n = 5 samples/group) and TEM (n = 5 samples/group) analysis to assess axonal regeneration.

### Histological and immunohistochemical analysis

Explanted samples were fixed in 10% formalin in PBS, cross-cut in the middle portion and retrogradely cut into 4 μm-thick serial sections after paraffin embedding. HE staining occurred according to routine protocols. In parallel, immunological characterization of the regenerated tissue inside the nerve conduits was carried out with the following antibodies diluted in PBS: anti-CD3 (polyclonal rabbit anti-human CD3, A 0452; Dako, Milan, Italy) diluted 1:500; anti-β-tubulin (polyclonal rabbit neuronal class III β-tubulin, PRB-435P; Covance, Princeton, NJ, USA); anti S-100 (polyclonal rabbit anti-S100, Z 0311; Dako) diluted 1:5000. Except for S-100, antigen unmasking was performed with 10 mM sodium citrate buffer, pH 6.0, at 90 °C for 10 min. The sections were then incubated for 30 min in blocking serum [0.04% bovine serum albumin (BSA; A2153, Sigma-Aldrich, Milan, Italy) and 0.5% normal goat serum (X0907, Dako)] to eliminate unspecific binding, and then incubated for 1 h at RT with the above primary antibodies. Primary antibody binding was revealed by incubation with anti-rabbit/mouse serum diluted 1:100 in blocking serum for 30 min at RT (Dako® EnVision + TM peroxidase, rabbit/mouse; Dako, Glostrup, Denmark) and developed in 3,3′-diaminobenzidine for 3 min at RT. Lastly, the sections were counterstained with haematoxylin. As a negative control, sections were incubated without primary antibodies.

### Morphological and morphometric assessment of nerve regeneration

Axonal regeneration was assessed by examining the ultrastructural features of the explants. Explanted samples were fixed in 2.5% in glutaraldehyde in 0.1 M PBS. Thereafter, each sample was divided in different portions to allow the evaluation of axonal regeneration at different levels; thus proximal, central and distal sections were considered (Fig. 5A). Controlateral sciatic nerve was also considered. Sections, post-fixed in 1% osmium tetroxide (Agar Scientific Elektron Technology - UK) in 0.1 M phosphate buffer, were dehydrated in a graded alcohol series and embedded in Epoxy resin. Semi-thin sections (0.5 µm) were cut with an ultramicrotome RMC-PTX PowerTome **(**Boeckeler Instruments, Arizona-USA) and stained with 1% Toluidine Blue. Images were acquired by using Leica DMR microscope (Leica Microsystems Wetzlar- Germany).

Ultrathin sections, 60 nm, were collected on 300-mesh copper grids, counterstained with 2% uranyl acetate and then with Sato’s lead. Specimens were observed by a Hitachi H-300 TEM.

Semi-thin cross-sections obtained from the proximal/central/distal portion of explants were stained with Toluidine Blue. Thereafter, photomicrographs were acquired using a Leica DMR microscope and imported into ImageJ image processing software (National Institutes of Health, Bethesda, MD) for blinded analysis. Hence, to quantitatively compare the regenerated nerves, morphometric analysis of Toluidine Blue-stained cross sections was performed on samples. Myelinated and unmyelinated axons were observed in cross sections from the proximal, central and distal portion of each tube. After measuring the area of each nerve, the nerve cross-section was divided into 5 quadrants, and 3 high-power fields (100x) of equal area from each quadrant were evaluated for myelinated and unmyelinated axons. Total axon number was then divided by the area sampled to calculate average axon density.

### Gait analysis

Prior to the surgical procedure and at 12 weeks from surgery, functional evaluation of peripheral nerve regeneration was assessed by the gait analysis. Rats with their feet stained with black ink were placed at the beginning of a gangway (100 cm long/10 cm wide) lined with white paper; the animals were allowed to walk up thereby leaving on the paper their footprints which were obtained and analyzed. The footprints of the non-operated side were also considered.

### Statistical analysis

Statistical analyses were performed by the Kruskal-Wallis test and Dunn’s multiple comparison test. Results were expressed as mean ± SD. *P* < 0.05 was considered to be statistically significant. Statistical calculations were carried out by Prism 3.0.3 (GraphPad Software, San Diego, CA).

## Electronic supplementary material

Supplementary figure S1. Histological and immunohistochemical analysis.

Supplementary video S2. Gait analysis in Sprague-Dawley rats at 12 weeks from surgery.
