# Supplementary material for: Partially oxidized polyvinyl alcohol conduitfor peripheral nerve regeneration
Source: Sci Rep. 2018 Jan 12;8:604. doi: 10.1038/s41598-017-19058-3 (PMC5766572; doi:10.1038/s41598-017-19058-3)
Supplement: Supplementary file 1 — Supplementary figure S1. Histological and immunohistochemical analysis. [file 41598_2017_19058_MOESM1_ESM.pdf]

Partially oxidized polyvinyl alcohol conduit for peripheral nerve regeneration

Elena Stocco, Silvia Barbon, Lucia Lora, Francesca Grandi, Leonardo Sartore, Cesare Tiengo, Lucia Petrelli, Daniele Dalzoppo, Pier Paolo Parnigotto, Veronica Macchi, Raffaele De Caro, Andrea Porzionato and Claudio Grandi

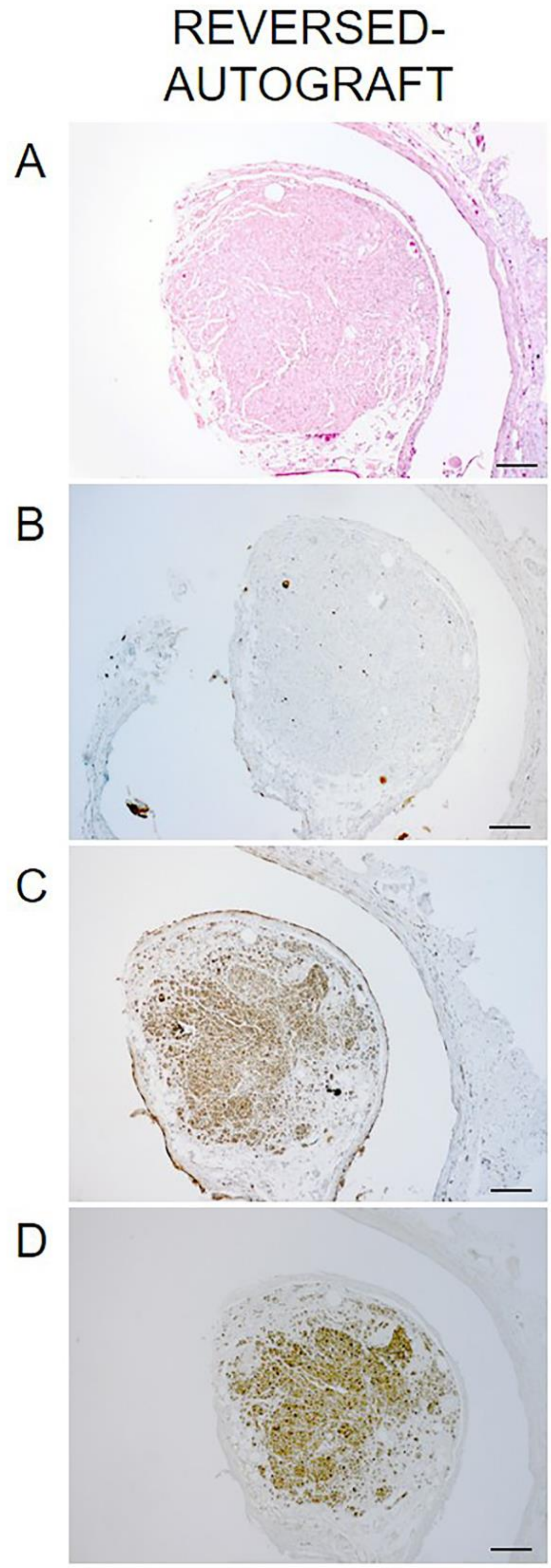

Supplementary figure S1. Histological and immunohistochemical analysis. Characterization of the central portion of explanted reverse-autograft by HE staining (A) and immunohistochemical anti-CD3 (B), anti-β-tubulin (C) and anti-S100 (D) reactions (scale bar = 100 µm).

Supplementary video S2. Gait analysis in Sprague-Dawley rats at 12 weeks from surgery. All animals showed a functional recovery but some differences were identifiable between the three experimental groups. In fact, unlike animals implanted with OxPVA, rats with nerve conduits in PVA and SF sometimes exhibited a gait characterized by paw-dragging (i.e. 00:00:44 min and 00:00:40 min for PVA; 00:00:09 for SF) limping (00:00:35 min for PVA) and spasms (00:00:31 min for PVA; 00:00:15 min for SF).
